# Duplication of a promiscuous transcription factor drives the emergence of a new regulatory network

**DOI:** 10.1038/ncomms5868

**Published:** 2014-09-10

**Authors:** Ksenia Pougach, Arnout Voet, Fyodor A. Kondrashov, Karin Voordeckers, Joaquin F. Christiaens, Bianka Baying, Vladimir Benes, Ryo Sakai, Jan Aerts, Bo Zhu, Patrick Van Dijck, Kevin J. Verstrepen

**Affiliations:** 1 Laboratory for Genetics and Genomics, Department M2S, Centre of Microbial and Plant Genetics (CMPG), KU Leuven, B-3001 Leuven, Belgium; 2 Laboratory for Systems biology, Vlaams Instituut voor Biotechnologie (VIB), B-3001 Leuven, Belgium; 3 Structural Bioinformatics, Center for Life Science Technologies (CLST), RIKEN, 230-0045 Yokohama, Japan; 4 Laboratory of Evolutionary Genomics, Centre for genomic regulation (CRG), 08003 Barcelona, Spain; 5 Genomics Core Facility, European Molecular Biology Laboratory Heidelberg (EMBL), 69117 Heidelberg, Germany; 6 Department of Electrical Engineering, STADIUS Center for Dynamical Systems, Signal Processing and Data Analytics, KU Leuven, B-3001 Leuven, Belgium; 7 iMinds Medical Information Technologies Department, KU Leuven, B-3001 Leuven, Belgium; 8 Molecular Microbiology and Biotechnology Section, KU Leuven, B-3001 Leuven, Belgium; 9 Department of Molecular Microbiology, VIB, B-3001 Leuven, Belgium

## Abstract

The emergence of new genes throughout evolution requires rewiring and extension of regulatory networks. However, the molecular details of how the transcriptional regulation of new gene copies evolves remain largely unexplored. Here we show how duplication of a transcription factor gene allowed the emergence of two independent regulatory circuits. Interestingly, the ancestral transcription factor was promiscuous and could bind different motifs in its target promoters. After duplication, one paralogue evolved increased binding specificity so that it only binds one type of motif, whereas the other copy evolved a decreased activity so that it only activates promoters that contain multiple binding sites. Interestingly, only a few mutations in both the DNA-binding domains and in the promoter binding sites were required to gradually disentangle the two networks. These results reveal how duplication of a promiscuous transcription factor followed by concerted *cis* and *trans* mutations allows expansion of a regulatory network.

## Abstract (web summary)

The molecular basis of transcriptional regulation evolution following gene duplication is poorly understood. Here the authors show how duplication of a promiscuous fungal transcription factor followed by concerted *cis* and *trans* mutations generates a novel regulatory network.

## Graphical abstract

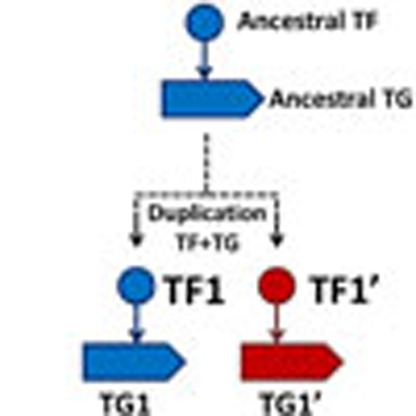

Gene duplication is believed to be an important driver of evolution because duplicated genes often diverge and evolve new functions^1^,^2^,^3^,^4^,^5^,^6^,^7^,^8^,^9^,^10^. Such gene duplication events occur relatively frequently, with at least 50% of prokaryotic genes and over 90% of eukaryotic genes estimated to have emerged by gene duplication^11^,^12^,^13^,^14^.

The emergence of new genes with novel functions might require reprogramming and/or extension of the regulatory network to ensure that the new paralogues are properly expressed^9^,^12^,^15^,^16^,^17^. Specifically, differential regulation of newly duplicated genes may be important to avoid ‘paralogue interference’, a situation where the duplicates interfere with each other’s function^17^. Gu *et al.*^15^ suggested a model of asymmetrical regulatory evolution of paralogue genes after the duplication, where the regulation of one gene copy evolves rapidly, while the other copy retains the ancestral expression profile. In keeping with this theory, several global genome-wide studies confirm that paralogues are often differentially expressed and show an increased rate of expression divergence. This likely reflects the need for cells to evolve specific regulatory programs for the ancestral and novel gene functions that emerge after the duplication event^8^,^12^,^15^,^16^,^18^,^19^,^20^,^21^,^22^.

While the number of studies demonstrating divergent transcriptional regulation of paralogues is increasing, few studies have investigated the molecular details underlying regulatory divergence. Some authors emphasize the importance of loss and gain of *cis*-regulatory elements in the evolution of paralogue regulation^19^,^22^,^23^,^24^,^25^,^26^. It has been demonstrated that whereas the number of *cis*-regulatory elements shared between two paralogues drops with their age, the total number of regulatory elements in their promoters remains the same, implying that a loss of regulatory motifs is compensated by gain of novel regulatory motifs^23^. Similarly, Ihmels *et al.*^22^ reported a large-scale loss of a specific *cis*-regulatory element from the promoters of dozens of genes following the whole-genome duplication in the yeast lineage. This event led to a major transcription network reprogramming and allowed optimization of anaerobic growth in *Saccharomyces cerevisiae*.

Apart from the importance of *cis* changes in the promoters of duplicated genes, changes in *trans*-acting factors (that is, transcription factors that regulate the paralogues) may also play a crucial role in the evolution of paralogue gene regulation. It has been shown that only 2–3% of the divergence in paralogue expression is explained by changes in *cis*-regulatory motifs^27^. Several studies further suggested that duplication of transcription factors may be an important mechanism that allows rewiring of existing regulatory networks or the development of new regulatory circuits^12^,^17^,^28^,^29^.

Teichmann and Babu^12^ proposed three basic scenarios of gene regulation after duplication that contemplates changes in both *cis* and *trans* regulatory elements. In the first scenario, both copies of the gene can stay under regulation of the same transcription factor, which is an expected outcome if the paralogues do not develop different functions, but are conserved because of dosage effects^7^,^11^,^30^. Alternatively, a newly duplicated paralogue can become a part of another (existing) regulatory network, for example after gaining a novel *cis*-regulatory element (that is, a transcription factor binding site that did not occur in the promoter of the ancestral gene). However, some cases of neofunctionalization, where one of the paralogues acquires a completely new function, may require a completely novel regulatory circuit. A third scenario therefore involves the generation of a new regulatory cascade by duplication and functional divergence of an existing transcription factor, so that each of the two paralogue target genes becomes regulated by one of the two newly duplicated transcription factors. However, the concerted duplication and evolution of a target and its transcription factor seems unlikely and intuitively requires a large number of concerted evolutionary events, except perhaps following a whole-genome duplication event^2^,^31^,^32^,^33^,^34^,^35^.

To investigate these scenarios, we focus on the regulation of the *MAL* genes in yeast. The *MAL* genes comprise three-gene subfamilies (*MALT*, *MALS* and *MALR*) that allow uptake and metabolism of various disaccharides, with each subfamily showing multiple duplication and neofunctionalization events^36^. The *MALT* subfamily encodes transporter proteins that allow active import of the sugars. Once inside the cell, the disaccharides are hydrolyzed by the MalS glycosidases. Some of the intracellular disaccharides are believed to bind the MalR regulator proteins, and these complexes activate the expression of the *MALS* and *MALT* genes^37^.

We have previously shown that the *MALS* genes in *S. cerevisiae* underwent several duplication events, with some of the paralogues gaining a novel hydrolyzing activity towards α 1–6 glycosidic bonds (found, for example, in isomaltose and palatinose), while other MalS paralogues retained the ancestral preference for α 1–4 glycosidic bonds (for example, in maltose)^7^,^36^. Similarly, the MalT transporters also underwent duplication and functional divergence, with some of today’s paralogues importing α 1–6 substrates, while others maintained the ancestral selectivity for α 1–4 glycosides^36^.

Here we investigated how the regulation of the *MALT* and *MALS* genes evolved after their duplication and functional divergence. We show that the present-day *S. cerevisiae MAL* genes are regulated specifically; that is, the palatinose-specific genes are activated only in response to palatinose-like sugars, whereas the maltose-specific paralogues are only activated by maltose-like sugars. We demonstrate that this specific regulation of the two paralogue groups became possible because the ancestral transcription factor MalR that regulated the ancestral *MALS* and *MALT* genes also underwent duplication. One new MalR paralogue activates the expression of novel *MALS* and *MALT* responsible for palatinose utilization, while the other MalR paralogue activates maltose-specific target genes. Furthermore, we establish a mutational path that explains how the differential regulation of both classes of paralogue target genes could have evolved without suffering from paralogue interference. Together, our results provide a detailed molecular view of how gene duplication can result in the emergence of a new transcriptional network.

## Results

### The *MAL* regulatory network allows specific regulation

The genome of the laboratory strain *S. cerevisiae* KV5000 harbours two functional *MALT* (transporter) and four functional *MALS* (maltase/isomaltase) genes. Following duplication, the activity of the MalS paralogues diverged, with Mal12 and Mal32 showing activity towards α 1–4 glycosides like maltose, and Ima1 and Ima5 hydrolyzing α 1–6 glycosides like isomaltose and palatinose. The two MalT paralogues show a lower degree of specialization, with Mal31 exclusively transporting α 1–4 glycosides, and Mal11 transporting both α 1–4 and α 1–6 disaccharides^7^,^36^.

**Figure 1:**
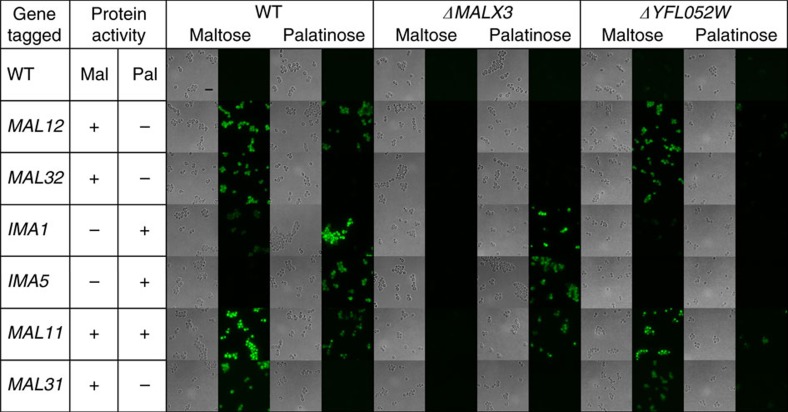
Maltose- and isomaltose-specific genes are differentially regulated. Representative brightfield and fluorescence microscopy images of yeast cells with various *MALS* or *MALT* genes fluorescently tagged are shown for wt cells (**a**) and strains carrying deletions of genes encoding transcriptional regulators (**b:** *MALX3* and **c:** *YFL052W*). Cells were grown in presence of either palatinose (α 1–6 disaccharide) or maltose (α 1–4 disaccharide) as indicated above the pictures. Gene names are listed in the first column, and protein activities towards the two types of sugars (maltose or palatinose) are indicated in the second and third columns. Scale bar is included in the upper left image and equals 10 μm. The experiment was repeated at least three times.

We first investigated whether expression of different *MALS* and *MALT* genes is regulated specifically by the sugar they show activity for. We therefore fluorescently tagged each of the four *MALS* and two *MALT* target genes in the wild-type (wt) strain and evaluated their expression in medium containing either maltose (α 1–4 disaccharide) or palatinose (α 1–6 disaccharide) using fluorescence microscopy. Figure 1a shows that the *MAL* gene regulation in *S. cerevisiae* is specific: most of the genes are only activated in presence of their respective substrate sugars (maltose or palatinose), and not in presence of the sugar for which they do not show activity. One notable exception is the *MAL12* hydrolase gene, which shows activity towards α 1–4 disaccharides, but also seems to be activated by the α 1–6 disaccharide palatinose. However, this gene shares a bidirectional promoter with the *MAL11* transporter gene, which transports both types of disaccharides. Hence, the aspecific activation of *MAL12* in palatinose may be a consequence of the need to activate *MAL11*, which encodes the only transporter that allows uptake of α 1–6 disaccharides like palatinose.

We have demonstrated previously that the *MALR* transcription factor genes *MALX3* and *YFL052W* are crucial for growth on maltose and palatinose, respectively^36^. To further investigate which of these transcriptional regulator genes is responsible for activation of which target gene(s), we deleted these *MALR* genes and investigated the effect on *MALT* and *MAL*S activation. Deletion of *MALX3* (Fig. 1b) abolishes the expression of the maltose-specific hydrolase genes *MAL12* and *MAL32*, and the maltose-specific transporter gene *MAL31*. Moreover, the MAL11 gene encoding the promiscuous transporter capable of transporting both α 1–4 and α 1–6 disaccharides is also no longer expressed in the presence of maltose, even though it is still activated in palatinose. Expression of *IMA1* and *IMA5* encoding α 1–6 hydrolases is not affected by deletion of *MALX3.* By contrast, deletion of the second *MALR* gene, *YFL052W*, abolishes expression of *IMA1* and *IMA5* (Fig. 1c), while the maltose-induced expression of genes encoding α 1-4-specific proteins (*MAL12*, *MAL32* and *MAL31*) and the promiscuous *MAL11* is not affected.

### Different MalR regulators bind different DNA sites

The previous results demonstrate that the *MALS* and *MALT* genes are regulated by two distinct regulatory networks, governed by different MalR transcription factors in response to different disaccharides. The limited crosstalk between the two regulatory networks suggests that the maltose- and palatinose-specific MalR regulators bind different DNA-binding sites. To test this, we determined the DNA-binding sites of the palatinose-specific regulator Yfl052w using the chromatin immunoprecipitation (ChIP)-exo technique^38^, and compared these to the known binding sites of the maltose-specific regulator Malx3 (ref. 39). The ChIP-exo analysis supports the results reported in Fig. 1 and indicates that both transcription factors bind different sites. Specifically, when the α 1–6 disaccharide palatinose is present, Yfl052w binds the promoter regions of palatinose-specific genes (*IMA1*, *IMA5* and *YFL052W*) and the *MAL11* promiscuous transporter, but not the promoters of maltose-specific genes (Supplementary Fig. 1). Instead, these maltose-specific genes are known to be bound by Malx3 in the presence of maltose^39^.

In addition, two novel noncanonical targets of Yfl052w were identified in the ChIP-exo experiment—*YHR210C* and *YJL217W* (Supplementary Fig. 1). Both of these genes seem to have no role in α-disaccharide metabolism and they do not show sequence similarity with *MAL* genes. Deletion of any of the two does not change cell growth or expression patterns of the other *MAL* genes (*IMA5* and *MAL32*) in palatinose or in maltose (Supplementary Fig. 2).

**Figure 2:**
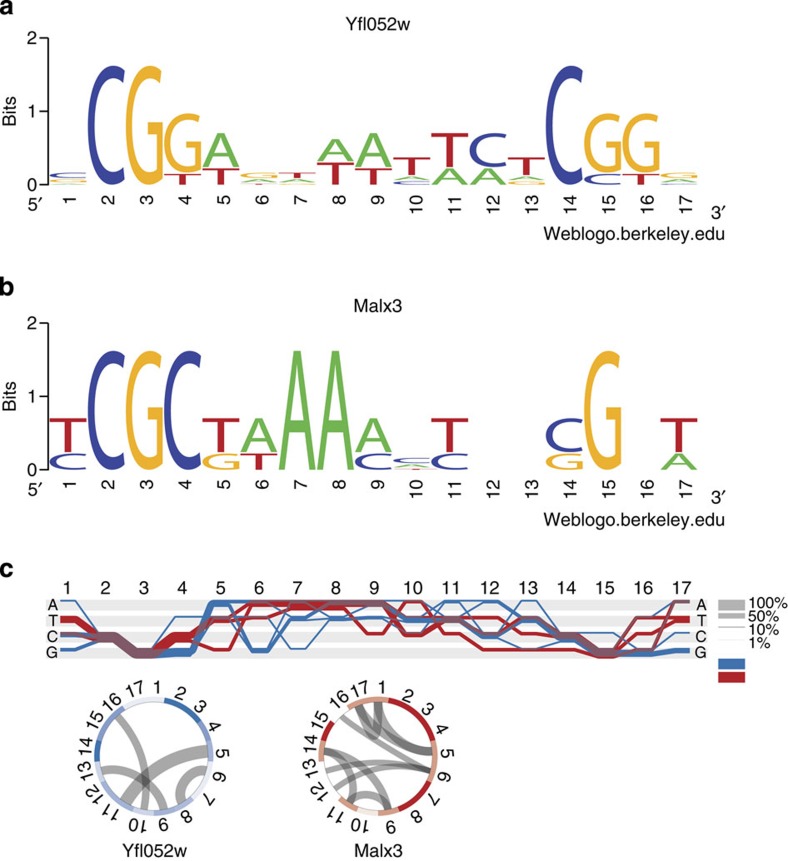
Different DNA-binding specificity of different MalR transcription factors. (**a**) Sequence logo of Yfl052w DNA-binding site CGG(9N)CGG. (**b**) Sequence logo of Malx3 DNA-binding site CGC(9N)CGN. (**c**) Sequence Diversity Diagram conveying differences and similarities between Yfl052w (blue) and Malx3 (red) binding sites. Regions where two groups overlap are shown in purple. Thickness of the line shows the relative proportion of the aligned sequences. Positions that differentiate two groups are identified by a separation of blue and red lines. The rings below are basic heatmaps in circular layout showing the information content of the positions. The more saturated the colour, the higher the information content, thus indicating conserved regions. Mutual information (MI) represents covariance of positions and is shown as grey lines inside the circles. High MI indicates the dependency of one position on another.

**Figure 3:**
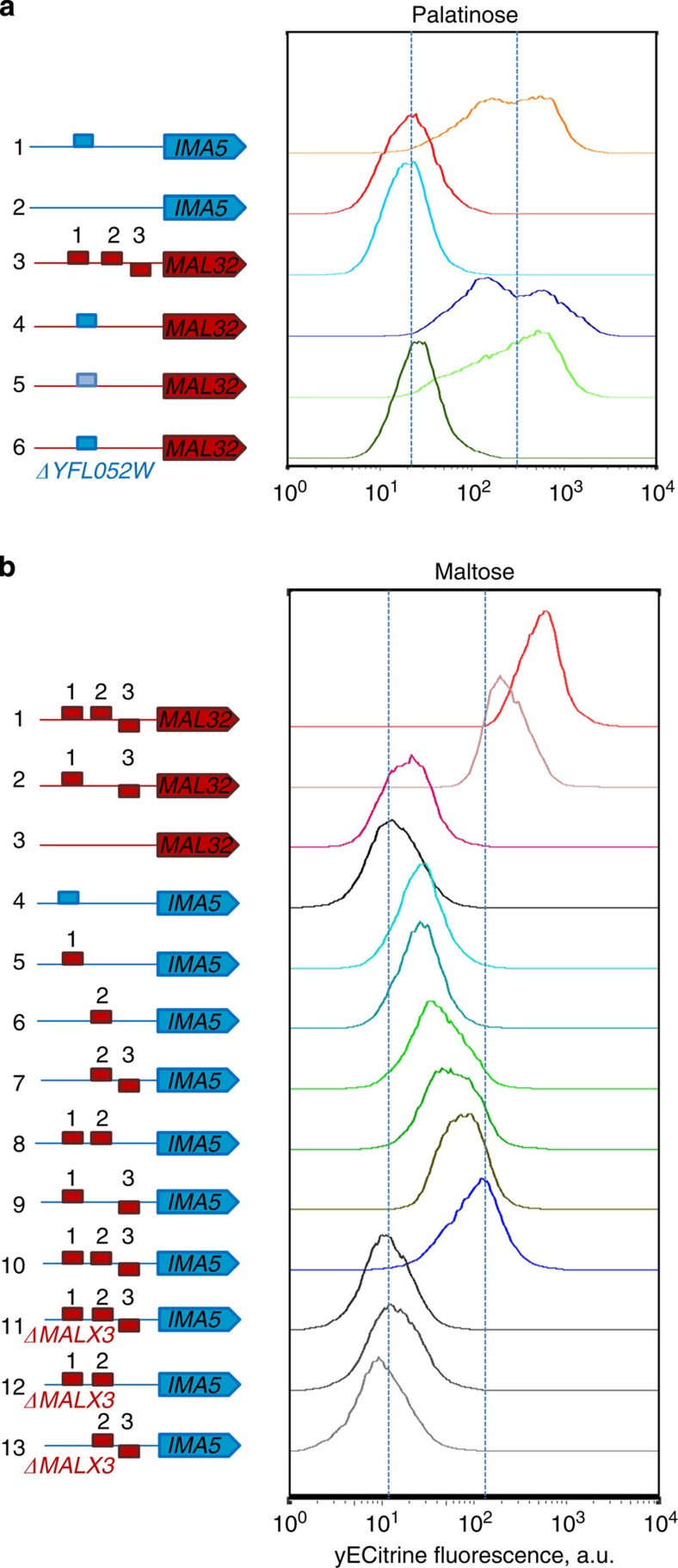
Maltose- and palatinose-specific MalR regulators have different DNA-binding sites. Representative flow cytometry histograms of populations of fluorescent reporter strains carrying different types of DNA-binding sites in the promoters of a maltose-specific gene *MAL32* (red block arrow), or a palatinose-specific *IMA5* (blue block arrow), grown in maltose or palatinose. CGG-containing binding sites found in promoters of palatinose-specific genes are depicted as blue rectangles, CGC-containing DNA-binding sites found in promoters of maltose-specific gene are depicted as red rectangles. (**a**) DNA-binding specificity of the palatinose-specific regulator Yfl052w. (1) A fluorescently tagged *IMA5* gene under its native promoter shows a normal level of *IMA5* expression activated by Yfl052w in palatinose. (2) Deletion of the Yfl052w binding site abolishes the expression of *IMA5.* (3) A fluorescently tagged maltose-specific *MAL32* under its native promoter shows no expression on palatinose and is used to quantify autofluorescence. Introduction of a Yfl052w binding site from the promoter of *IMA5* (4) or *IMA1* (5) in front of the fluorescently tagged *MAL32* gene makes the expression of this gene responsive to palatinose. (6) Deletion of *YFL052W* abolishes the expression of the *MAL32* gene from (4). (**b**) DNA-binding specificity of the maltose-specific regulator Malx3. (1) A fluorescently tagged *MAL32* under its native promoter shows a normal level of *MAL32* expression activated by Malx3 in maltose. (2) Deletion of one Malx3 binding site decreases the expression levels of fluorescently labelled *MAL32*. (3) Deletion of all three Malx3 binding sites abolishes the expression of *MAL32*. (4) A fluorescently tagged *IMA5* gene under its native promoter is used to show autofluorescence. Introduction of one (5), (6), two (7)–(9) or three (10) Malx3 binding sites in the promoter region of a fluorescently tagged *IMA5* gene makes the expression of this gene responsive to maltose, with increasing number of binding sites resulting in higher expression levels. (11)–(13) Deletion of the maltose-specific regulator *MALX3* abolishes the expression of strains from (7), (8) and (10). Each experiment was repeated at least three times with two biological replicates.

**Figure 4:**
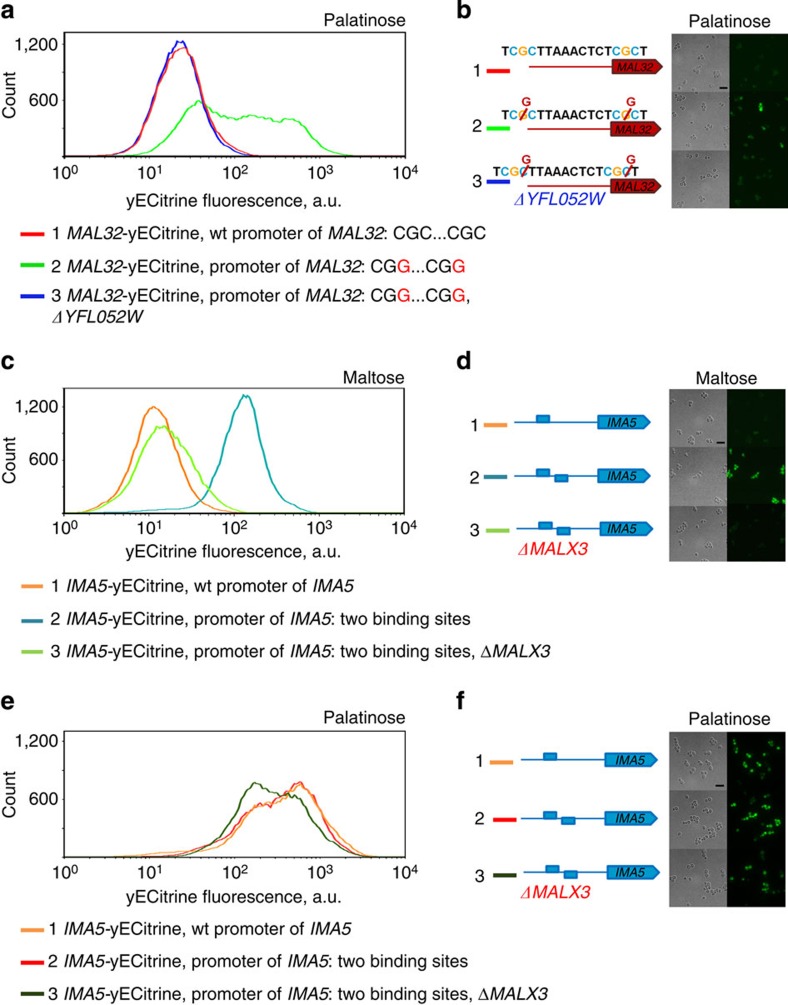
Yfl052w and Malx3 regulators show different DNA-binding specificity. (**a**) Two point mutations in a maltose-inducible promoter yield a palatinose-inducible promoter. (1) Histogram of the fluorescence signal of a strain with a yECitrine-tagged *MAL32* gene. This maltose-specific reporter gene shows no expression in palatinose and can be used to estimate the background fluorescence levels. (2) Single nucleotide C to G substitution in both CGC motifs in the upstream Malx3 binding site of the of *MAL32* promoter leads to expression of this gene in palatinose. (3) Deletion of palatinose-specific regulator Yfl052w abolishes the expression of the mutant promoter. (**b**) Fluorescence microscopy images of the same strains reported in **a.** (**c**) Increasing the number of CGG-containing binding sites in a palatinose-inducible promoter yields a promoter that is responsive to maltose. (**d**) Representative fluorescence microscopy images of the strains shown in **c** in maltose. (**e**) Increasing the number of CGG-containing binding sites does not affect the expression of the palatinose-inducible gene *IMA5* in palatinose. (**f**) Representative fluorescence microscopy images of the strains shown in **c** in palatinose. Scale bar is included in the upper left image of **b**,**d**,**f** and equals 10 μm. Each experiment was repeated at least three times with two biological replicates.

The MalR regulators belong to a family of fungal Zn-finger transcription factors, which typically bind short three-nucleotide CG-rich motifs separated by a spacer of fixed length^40^. Figure 2 shows that the DNA-binding site of the palatinose-specific Yfl052w regulator is very similar to that of the maltose-specific Malx3 regulator. Specifically, the Yfl052w binding site consists of two CG**G** motifs separated by a nine nucleotide (nt) AT-rich spacer (Fig. 2a), while Malx3 DNA-binding sites contain a CG**C** motif, a nine nt spacer and a CGN motif (Fig. 2b). To confirm the binding sites of the different MalR regulators (Malx3 and Yfl052w), we first deleted the binding sites in a strain carrying a fluorescent reporter fusion of a maltose-specific target gene (*MAL32*) and in a strain carrying a reporter for a palatinose-specific gene (*IMA5*). In both cases, deletion of the respective binding sites abolished induction of the target gene by its respective substrate sugar (Fig. 3a: lines 1 and 2 and Fig. 3b: lines 1, 2 and 3). Moreover, replacing one binding site with the other switches the sugar-specificity of the promoters as well as the specific transcription factor needed to activate the reporter gene (Fig. 3), further suggesting that these slightly different binding sites separate the two regulatory circuits. Finally, to further confirm that this single-nucleotide difference is indeed responsible for the different binding of both classes of transcription factors, we introduced a double point mutation in the promoter region of *MAL32* gene, so that both CG**C** motifs in the Malx3 binding site were changed to CG**G** motifs. As shown in Fig. 4a,b, these mutations result in *MAL32* gene expression in presence of palatinose, and this effect was dependent on the *YFL052W* gene.

Apart from the one-nucleotide difference between the binding motifs, we also noticed that the promoters of maltose-specific genes (*MAL12*, *MAL32* and *MAL31*) and the promiscuous *MAL11* transporter always contain three Malx3 binding sites, while palatinose-specific genes (*IMA1*, *IMA5* and *YFL052W*) contain only one Yfl052w binding site. Interestingly, the results shown in Fig. 3 suggest that all three Malx3 binding sites are necessary to obtain full activation of the downstream gene, with one or even two binding sites only yielding partial activation.

### Two key mutations in Yfl052w alter its binding preference

**Figure 5:**
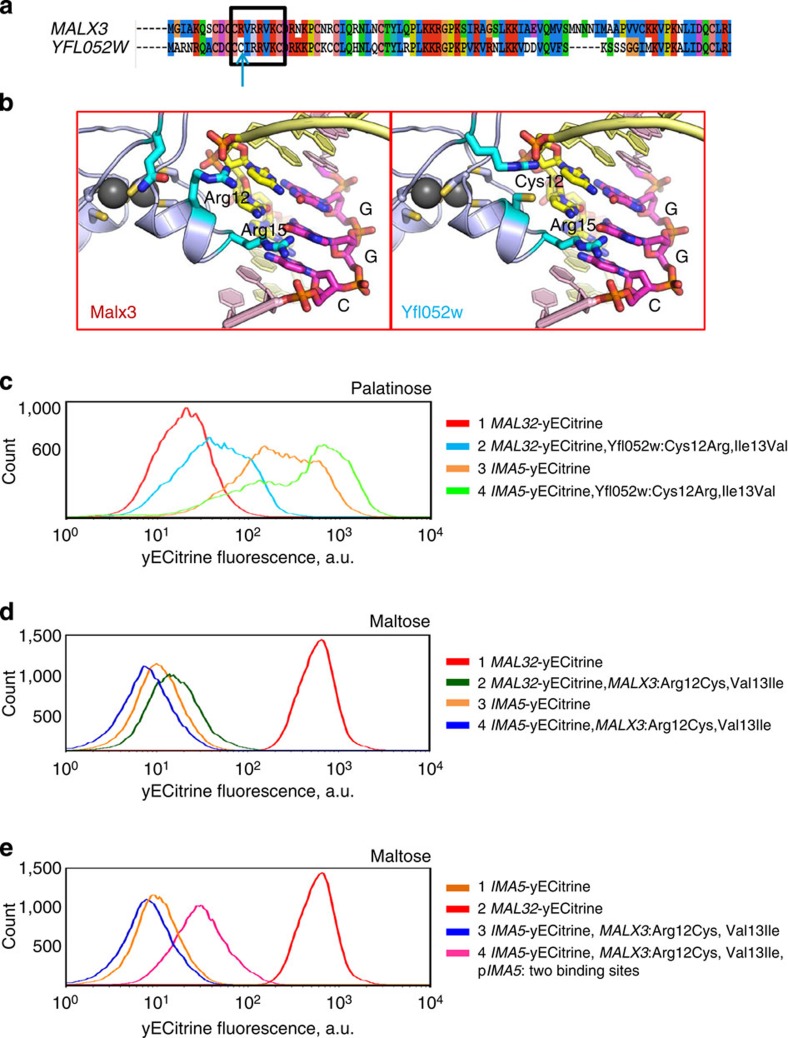
Differences in the DNA-binding domain of Malx3 and Yfl052w explain their different binding specificity. (**a**) Alignment of the Malx3 and Yfl052w DNA-binding domains. Amino acids predicted to interact with the DNA-binding site are indicated with a black rectangle. The key position 12 that differs between Malx3 and Yfl052w is highlighted with a blue arrow. (**b**) Molecular modelling of the interaction between the Zn-finger domain and its DNA-binding site. Important base pairs are represented as yellow and magenta sticks, important amino acids are represented as blue sticks. The Arg15 is shared between both transcription factors and is responsible for the recognition of the G in the middle of the CGG binding motif. Arg12 in Malx3 does not take part in recognition of the CGG motif, but Cys12 in Yfl052w does interact with the DNA and is responsible for the preference for a G nucleotide in the third position of the motif. (**c**) A mutated version of the palatinose-specific Yfl052 activator (Cys12Arg and Ile13Val) is able to partly activate the *MAL32* promoter in response to palatinose and also retains its capacity to activate the *IMA5* promoter. (**d**) A mutated version of the maltose-specific Malx3 activator (Arg12Cys and Val13Ile) is incapable to activate *MAL32* or *IMA5* in response to maltose. (**e**) A mutated version of the maltose-specific Malx3 activator (Arg12Cys and Val13Ile) is capable to partly activate an *IMA5* promoter containing an additional Yfl052w binding site. Each experiment was repeated at least three times with two biological replicates.

Next, we turned our attention to the MalR transcription factors. To determine which amino acid residues of palatinose-specific regulator Yfl052w could be responsible for its altered DNA-binding specificity, we modelled the three-dimensional structures of Yfl052w and Malx3 in complex with their DNA-binding sites (Fig. 5). Our model suggests that the amino acid in position 12 may explain the difference in DNA-binding preference between Malx3 and Yfl052w. In Malx3, position 12 is occupied by Arg, which is not involved in the base pair recognition, but does interact with the negatively-charged phosphate backbone of DNA. In Yfl052w, position 12 is taken by a Cys residue, which in contrast to the Arg residue in Malx3 does interact with the bases of the DNA-binding motif and specifically requires the presence of a G in the third position of CGG motif (Fig. 5b). In addition, Val in position 13 in Malx3 is substituted with Ile in Yfl052w. Both Val and Ile provide the hydrophobic environment required for amino acid in position 12. However, the Cys residue needs to be accompanied by a more hydrophobic amino acid, which might explain the exchange of Val (hydrophobicity index 79) to Ile (100).

To confirm whether the preference of Yfl052w to CGG motifs is indeed dictated by the two residues mentioned above, we mutated Cys12 in Yfl052w to Arg, and Ile13 to Val and tested the binding specificity of this mutated Yfl052w. As shown on Fig. 5c, the mutated Yfl052w is able to partially activate the expression of maltose-specific gene *MAL32* in palatinose. Moreover, the fluorescence profile of these cells resembles that of wt cells with fluorescently tagged *IMA5*, which is also activated by Yfl052w. Interestingly, the mutated Yfl052w apparently also still activates its natural target promoter that drives *IMA5*.

Similarly, we introduced Arg12 to Cys and Val13 to Ile mutations in the Malx3 regulator. This mutated Malx3 regulator can no longer activate expression of its natural target *MAL32* promoter (containing CGC motifs), nor can it activate the palatinose-specific *IMA5* gene (Fig. 5d). However, we showed earlier that Malx3 requires several DNA-binding sites to activate expression of its target genes (Fig. 3b). Indeed, when a second CG**G**-containing binding site was introduced in the promoter of a fluorescently labelled *IMA5* reporter strain, the mutated Malx3 was able to activate the expression of *IMA5* in maltose, which is in keeping with the observation that Malx3 requires multiple binding sites (Fig. 5e).

### Malx3 binds both CGC and CGG motifs

Structural modelling of Malx3 bound to DNA predicts that Malx3 is promiscuous and can bind both CG**C** and CG**G** motifs. This reduced binding stringency compared with Yfl052w is predicted to be a result of the Arg12 residue in the Malx3 binding domain, which allows both C and G in the third position of the binding motif. This prediction is supported by the observation that Yfl052w carrying a Cys12Arg substitution still activates expression of its natural target *IMA5* that carries CG**G** motifs in its promoter (Fig. 5c). On the other hand, the results shown in Fig. 1 demonstrate that Malx3 does not activate expression from CG**G**-containing promoters of palatinose-specific target genes *in vivo*. However, the results from Fig. 3b show that Malx3 requires the presence of more than one binding site to activate expression, and promoters of palatinose-specific genes only contain one MalR binding site. In other words, whereas Malx3 is able to bind both CG**G** and CG**C** motifs, Malx3 may not be able to activate CG**G**-containing palatinose-specific promoters because they only contain one binding site; whereas maltose-specific promoters contain multiple sites. To verify this hypothesis, we introduced a second CG**G**-containing Yfl052w binding site in the promoter region of the fluorescently labelled *IMA5* gene and measured the expression of this palatinose-specific gene in the presence of maltose. As shown in Fig. 4c,d, the introduction of an additional binding site leads to the activation of *IMA5* gene expression by maltose to a level similar to its normal activation in palatinose, even in the absence of the Yfl052w transcription factor that normally activates *IMA5*.

Together, these results suggest that specific binding of Yfl052w to the promoter regions of palatinose-specific target genes is determined by the presence of CGG motifs. Promoters of maltose-specific genes contain CGC motifs and thus cannot be bound by Yfl052w because the Cys12 residue in the DNA-binding domain of this regulator prevents binding CGC sites. On the other hand, Malx3 is capable of binding both types of motifs (CGG and CGC), but requires the presence of several binding sites in the same promoter region to yield full gene activation. This prevents Malx3 from activating expression of palatinose-specific genes, which carry only one MalR binding site in their promoters.

### Evolutionary model of divergence of two regulatory networks

Taken together, our results uncover the mechanistic details underlying the emergence of two separate and specific regulatory circuits, one regulating maltose metabolism and the other regulating isomaltose and palatinose metabolism. We next wanted to establish the likely evolutionary path from the ancestral, pre-duplication circuit to the present-day situation.

The common ancestor of extant yeast species only had one copy of each of the three types of *MAL* genes (*MALS*, *MALT* and *MALR*)^7^,^36^. In some species, including *S. cerevisiae*, the *MAL* genes underwent several duplication events. In other species, like *L. elongisporus*, the *MAL* genes were not duplicated and the ancient, simple three-gene network seems to be preserved (Supplementary Fig. 3). Moreover, the activity of the MalS protein of *L. elongisporus* resembles that of the pre-duplication ancestral enzyme and can hydrolyze both maltose- and palatinose-like disaccharides^7^. This suggests that its MalR regulator may be able to activate the expression of both the promiscuous MalS and the promiscuous MalT in presence of any of the two types of sugars. To test this hypothesis, we compared mRNA levels of *MALS* and *MALT* genes in *L. elongisporus* cells grown on either maltose, palatinose or glucose. As shown in Supplementary Fig. 4, expression of *MALT* and *MALS* is indeed activated in presence of both maltose and palatinose, but not glucose.

Next, we investigated the timing of the duplication and divergence of the *MALS* and *MALR* genes (Supplementary Fig. 3). We have previously shown that the *MALS* genes duplicated and acquired a novel function at least at the branching of *Kluyveromyces thermotolerans*^7^. A similar analysis shows that the functional diversification of *MALR* genes happened later in the evolution, around the branching of *S. bayanus* from the *Saccharomyces* clade. Specifically, the genome of *S. bayanus* contains only one *MALR* of the promiscuous *MALX3* type (Arg in the position 12) and lacks the palatinose-specific Yfl052w-like regulator (containing a Cys12Arg mutation), which is present in *S. cerevisiae*, *S. paradoxus*, *S. mikatae* and *S. kudriavzevii*. Expression analysis shows that the regulation of different *MALS* genes in *S. bayanus* is not specific, that is, maltose-specific and palatinose-specific *MALS* genes are equally activated in presence of maltose and palatinose, but not glucose (Supplementary Fig. 5). Furthermore, the nucleotide sequences of the *MALR* binding sites in the genomes of *S. bayanus*, *S. mikatae*, *S. paradoxus* and *S. kudriavzevii* are remarkably conserved. Similar to the sites in *S. cerevisiae,* they fall into two classes: CGG and CGC containing (Supplementary Data 1). Analogous to *S. cerevisiae*, in *S. mikatae, S. paradoxus* and *S. kudriavzevii* (that is species in which the *MALR* regulator as well as the *MALS* genes has duplicated and diversified), CGG-containing sites are found in the promoter regions of palatinose-specific genes, and CGC-containing sites are situated upstream of homologues of maltose-specific genes. By contrast, in *S. bayanus* (which contains multiple *MALS*, but only one Arg12-type *MALR*), CGG and CGC sites are seemingly randomly distributed in the promoters of homologues of the different maltose- as well as palatinose-specific genes (Supplementary Data 1), which agrees with the nonspecific regulation of these genes in *S. bayanus*.

**Figure 6:**
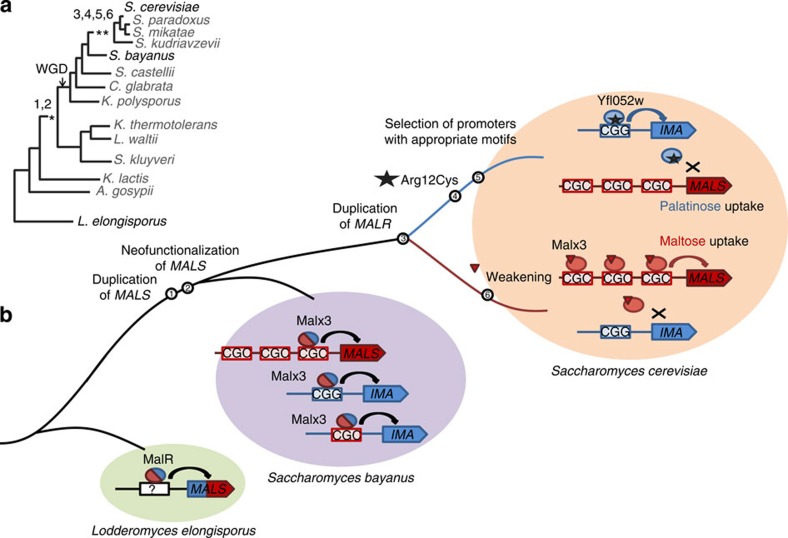
Possible evolutionary mutational path of *MAL* regulatory network diversification. (**a**) Simplified phylogenetic tree of the fungal lineage. The numbers correspond to the key evolutionary events listed in **b**. WGD denotes the documented whole-genome duplication event in the fungal lineage. (**b**) Likely evolutionary path of the *MAL* regulatory network. The path starts from the common ancestor of *L. elongisporus*, *S. bayanus* and *S. cerevisiae* and ends at the modern day *S. cerevisiae*. In the common ancestor of *L. elongisporus*, *S. bayanus* and *S. cerevisiae*, maltose and isomaltose enzymatic activities are not separated and coexist in a single ancestral MalS enzyme, which is regulated by the single promiscuous MalR regulator. In the common ancestor of *S. cerevisiae* and *K. thermotolerans*, the *MALS* genes duplicated and neofunctionalized (1, 2), so that both types of target genes (maltose and palatinose specific) are present and are regulated by one promiscuous Malx3-like transcription factor that has an Arg residue at position 12 allowing it to bind both CGG and CGC motifs. The regulation is not specific at this point, that is, palatinose- and maltose-specific genes are equally expressed in presence of their respective substrate as well as a nonspecific disaccharide (as it is in *S. bayanus*). Two separate regulatory circuits that appear around the deviation of *S. bayanus* from the *Saccharomyces* tree. The *MALR* gene is duplicated (3) and this duplication event is followed by two single-nucleotide mutations in the first positions of the Arg12 and Val13 codons, changing these to Cys and Ile in one of the paralogues (4), thus preventing it from binding CGC motifs in the promoters of maltose-specific genes. Analysis of genomes that carry only one type *MALR* gene suggests that in the ancestral yeast CGG and CGC motifs were randomly distributed among maltose- and palatinose-specific genes. This implies that these binding sites needed to change in concert with the mutations in the *MALR* paralogues, so that palatinose-specific genes only contain one CGG site, and maltose-specific genes contain three CGC motifs so that they can still be activated by the weakened Malx3 paralogue (5, 6).

Together, these observations reveal that the *MAL* gene network evolved as depicted in Fig. 6. In this model, duplication and functional divergence of the *MALS* genes already happened in the common ancestor of *K. thermotolerans* and *S. cerevisiae* (Fig. 6, events 1 and 2), but these genes were still controlled by one promiscuous MalR regulator that resembled today’s *S. bayanus* Malx3 protein (which has an Arg residue in position 12). Similar to the present-day Malx3 regulator, the ancestral regulator was able to bind both CGG and CGC motifs and induced the expression of both maltose- and palatinose-specific genes in presence of both types of sugars. The promoters of these genes probably did not yet diverge, with both CGC and CGG motifs present upstream of maltose as well as palatinose-specific genes similar to the genome of present-day *S. bayanus*. Yfl052w-like regulators most probably first appeared later in the evolution as a result of duplication (Fig. 6, event 3) and subsequent mutations, including the key Arg12Cys mutation (Fig. 6, event 4). This mutated Yfl052w-like paralogue can no longer bind the CGC motifs, which are selected for in the promoters of maltose-specific genes. By contrast, the Malx3-like regulator evolves a weaker activity, so that it loses the ability to activate expression of palatinose-specific genes, which are selected to have only one CGG-containing binding site, while retaining the ability to activate maltose-specific promoters that contain three CGC binding sites (Fig. 6, events 5 and 6).

## Discussion

Several studies have investigated the regulatory divergence between species on a genome-wide level^8^,^12^,^18^,^20^,^22^,^41^,^42^,^43^,^44^,^45^,^46^,^47^,^48^. Together, these studies show that changes in gene regulation occur frequently and are important drivers of functional and morphological evolution^26^,^46^,^47^. This is especially true for the evolution of the regulation of newly duplicated genes. Since paralogues often evolve different functions, these functionally diverged duplicates may need to be regulated independently. However, despite the importance of the evolution and divergence of gene regulation, the exact molecular mechanisms and mutational pathways that lead to the emergence of such novel regulatory networks remain largely unknown.

Our results show how duplication of a promiscuous transcription factor and its target genes led to the development of two separate regulatory networks, with one paralogue of the transcription factor regulating a set of target genes involved in maltose uptake and metabolism, and another regulating target genes responsible for palatinose consumption. Specifically, we find that only two point mutations in the promoter regions of the target genes, combined with two single-nucleotide mutations in the DNA-binding domain of the transcription factor paralogues are sufficient to ensure that each transcription factor paralogue specifically activates its target promoters, without interfering with the regulation of the target genes of the other paralogue.

While the predominant opinion in the field is that evolution on the regulatory level precedes the actual changes in the protein sequence of the target genes^8^,^10^,^18^,^21^,^22^,^25^,^49^, our data indicate that the opposite is also possible. It seems likely that the pre-duplication ancestral *MAL* gene regulatory network was very simple and resembled the network in present-day *L. elongisporus* (Supplementary Fig. 3). The *L. elongisporus* MalR regulator is promiscuous and activates expression of a (bifunctional) MalS hydrolase and a transporter in response to either maltose or palatinose. Several duplication events of *MALS* genes followed by optimization of either maltase or palatinase activity in different paralogues led to emergence of two functional classes of MalS hydrolases in *S. cerevisiae*^7^. Interestingly, our analyses suggest that the specialization of palatinose-specific MalR regulators and the separation of the two regulatory networks likely occurred after the neofunctionalization of *MALS* target genes, around the branching of the *S. bayanus* and *S. cerevisiae* clades (Supplementary Fig. 3). The functional divergence of the MalS enzymes generated a situation where it became beneficial for the cells to regulate each of the MalS enzymes separately, so that each enzyme is only activated by its proper substrate and paralogue interference is avoided. In keeping with this hypothesis, we have previously shown that activation of *MAL* genes in conditions where they are not required comes at a considerable fitness cost^50^.

The evolutionary path described in our study highlights how promiscuity (or limited binding site specificity) may increase the ‘evolvability’ of transcription factors by facilitating the emergence of distinct regulatory modules. Indeed, following a duplication event, successive mutations may allow a gradual increase in the specificity of the newly duplicated transcription factor paralogues and promote a smooth emergence of two independent regulatory circuits while avoiding misregulation of the target genes during this process (that is, a so-called ‘fitness valley’ is avoided). Importantly, only two key mutations in the Zn-finger DNA-binding domain are needed to increase the binding specificity of the Yfl052w paralogue. Together, these observations reveal how the seemingly unlikely model for the emergence of a new regulatory module through duplication of a transcription factor proposed by Teichmann and Babu^12^ can in fact really occur. However, in contrast with the theory that regulation evolves asymmetrically, with only the regulation of the new function diverging from that of the ancestral function^15^, we find that in this case the evolution of two separated regulatory networks depends on concerted changes in both networks (one regulating α 1–4 glycoside metabolism and the other regulating α 1–6 glycoside metabolism).

Interestingly, the importance of promiscuity also emerges from other studies that investigate the evolution of other proteins, such as enzymes and receptors. For example, the ancestral pre-duplication maltase showed activity towards both α 1–4 glycosides like maltose, but also a (trace) activity for α 1–6 glycosides such as isomaltose. Similarly, promiscuity has also been shown in other pre-duplication ancestral genes^2^,^51^,^52^. Hence, whereas promiscuity and ‘side activities’ are often regarded as imperfections, they are emerging as crucial factors that promote ‘evolvability’ because the side activities can be selected for and drive the evolution of a paralogue after duplication^53^.

It is especially interesting to compare our results to those reported in a recent elegant study by Baker *et al.*^17^ These researchers showed how duplication of the ancestral fungal Mcm1 transcription factor resulted in two paralogues that each evolved to regulate a subset of the original target genes. In contrast to the *MAL* gene system, the Mcm1 target genes were not duplicated, and duplication of the Mcm1 factor did not lead to the development of a completely separate regulatory circuit, but rather resulted in subfunctionalization and rewiring of the existing network, with the two paralogues diverging to regulate a subset of the original target genes. Moreover, whereas the MalR paralogues evolved different DNA-binding sites, the Mcm1 paralogues primarily evolved specificity through mutations that restrict their interaction with other transcription factors (Arg81 and Matα1), showing that this is a possible alternative route to rewire networks.

At first, the scenario of the evolution of the *MAL* genes, where both a transcription factor and its targets are duplicated, might seem rare. However, such situations may occur relatively frequently, either independently, or as a result of whole-genome duplication events^8^,^19^,^54^,^55^. Interestingly, the fungal lineage shows evidence for at least one whole-genome duplication event^34^,^56^ (see Supplementary Fig. 3). Several authors suggested that post whole-genome duplication networks may undergo functional partitioning, with the paralogues forming two independent subnetworks, which resembles the *MAL* gene scenario^8^,^19^. Moreover, a number of studies report that after the whole-genome duplication, the regulatory genes are preferentially retained compared with other functional classes of genes^2^,^31^,^32^,^33^,^35^. However, in the case of the *MAL* genes, the observed duplication events do not coincide with the reported whole-genome duplication event and instead seem to be the results of (multiple) independent duplications of smaller chromosomal regions. Interestingly, Lynch and Katju^57^ proposed that such small-scale duplications might result in misregulation of the duplicated gene when the respective promoter region is not duplicated. However, in the case of the MAL genes, the duplication events probably included the regulatory regions of the duplicated genes, as well as a (subsequent) duplication of the gene encoding the Mal regulator. While such events may be more rare than those associated with whole-genome duplications, they may occur relatively frequently in subtelomeric regions^36^. Moreover, many transcription factors show some level of promiscuity in their recognition of target sites^58^,^59^. As detailed above, such promiscuity may greatly facilitate the expansion and rewiring of transcriptional networks. Hence, whereas the molecular details may differ, the general themes uncovered in this study of the *MAL* regulatory circuit may be representative for a large number of similar events throughout the tree of life.

## Methods

### Strain construction

A complete list of strains and plasmids used in this study is listed in Supplementary Data 2. The primers used to make and confirm these strains can also be found in Supplementary Data 2. All constructs were verified by Sanger sequencing and/or PCR.

### Microbial strains, plasmids and growth conditions

We showed earlier that the *S. cerevisiae* feral isolates RM11 (from a vineyard) and YJM789 (from an AIDS patient) as well as laboratory strain EM93, ancestral to S288c, can ferment maltose due to the presence of a *MALX3* regulator in their genomes^36^; whereas *S. cerevisiae* strain S288c lost its *MALX3* regulator together with the ability to grow on maltose. We re-introduced *MALX3* in the genome of *S. cerevisiae* S288c to restore its ability for growth on maltose and named the resulting strain KV5000. Therefore, KV5000 represents a reconstituted wild-type *S. cerevisiae* strain, and for this reason, we refer to this strain as the wt strain.

Yeast cultures were grown in rich yeast extract and peptone (YP) media consisting of 2% peptone (Difco), 1% yeast extract (Difco) and 2% sugar (Sigma-Aldrich) at 30 °C in a rotating wheel or shaking incubator. The sugars used in this study were purchased to their highest available purity and were filter-sterilized before adding to rich media. Plasmid sets were obtained from EUROSCARF ( http://web.uni-frankfurt.de/fb15/mikro/euroscarf/) for reusable markers (Deletion Marker Plasmids) and overexpression/epitope tagging^60^. Plasmids were used as indicated by the manufacturer.

### Fluorescent microscopy imaging

Cell were pre-grown overnight in YP 2% glucose medium and then transferred to YP media supplemented with either palatinose (2%) or maltose (2%) for another 16 h. The acquisition of the images was done with the Optomorph software (version 1.0.2) in combination with a Nikon Eclipse Ti microscope equipped with a DL-604M-#VP camera (Andor technology). Images were processed and scaled with ImageJ software.

### Flow cytometry to measure gene expression levels

Cell were grown in YP medium supplemented with maltose (2%) or palatinose (2%) till the OD_600_=0.1. Fluorescent histograms were acquired using a BD Biosciences Influx flow cytometer with 488 nm laser coupled to 530–540 nm detector.

### RNA isolation and quantitative PCR

RNA was isolated with phenol/chloroform. Genomic DNA elimination and reverse transcription was performed using the QIAGEN QuantiTech Reverse Transcription kit according to manufacturer’s instructions. AB Power SYBR Green PCR master mix was used for quantitative PCR.

### Modelling

To investigate the difference between the MalR proteins, homology models were constructed. As there are no homologoes template structures available for the C-terminal domain, only the N-terminal DNA-binding region was investigated. Sequence comparison and fold recognition using Phyre2 indicated pdb entry 1D66 (Gal4-DNA complex) as the most suitable template^40^,^61^. All complexes were modelled using the homology implementation in the Molecular Operating Environment (Chemical Computing group, Montreal, Canada) with the implemented CHARMM force field in the presence of the 1D66 DNA structure^62^. Prior to modelling complexes, the base pair sequence of 1D66 sequence was adapted according to the MalR recognition motifs. Following the homology modelling incorporating the DNA structures the complex was optimized by steepest descent minimization in the presence of explicit water molecules.

### ChIP-exo

ChIP-exo was performed following the Pugh’s lab protocol^63^ by Peconics LLT, USA and independently repeated by EMBL GeneCore, Germany. KP54 strain with haemagglutinin-tagged Yfl052W was used for analysis. Untagged strain KP52 served as a control. DNA–protein complexes were precipitated using the Roche Anti-haemagglutinin high affinity rat monoclonal antibody (clone 3F10).

### Chip-exo data analysis

After Illumina sequencing, low quality reads (q<30) and adaptor sequences were first trimmed by using Trim Galore! ( http://www.bioinformatics.babraham.ac.uk/projects/trim_galore/). Resulting reads were then mapped to the *S. cerevisiae* reference genome (S288C, R64-1-1) by BWA version 0.7.4 (ref. 64) with default parameters. BAM-formatted files were then generated using samtools version 0.1.18 (the sequence alignment/map format and SAMtools) and further sorted following chromosome order by picard version 1.100 ( http://picard.sourceforge.net). Genome wide Event finding and Motif discovery (GEM) version 2.41 was used to detect the positive peak and motif discovery^65^. Default parameters were used, except—k_min was set to 5, -- k_max was set to 15, —s was set to 10,000,000 and —smooth was set to 3. After comparing the experimental sample with the control, the coordinate from resulted peaks were then visualized by using IGV 2.3 to confirm the accuracy^66^.

### Bioinformatics analyses

Genomic sequences of *S. cerevisiae* S288C, *S. mikatae* IFO 1815 (AABZ00000000.1), *S. kudriavzevii* IFO 1802 (AACI00000000.3), *S. paradoxus* NRRL Y-17217 (AABY00000000.1), L. elongisporus NRRL YB-4239 (AAPO00000000.1) were downloaded from the National Center for Biotechnology Information. *S. bayanus* CBS7001 genomic sequence is downloaded from www.SaccharomycesSensuStricto.org. Homologues of different MalR, MalS and MalT genes were identified with local BLAST+ suite. Upstream regions (−800;0 nt from the translation start) of found homologues were used to determine possible MalR DNA-binding sites. Protein alignment and *K*s score calculations were performed using MEGA software (version 5.2).

## Authors contributions

K.J.V. and K.P. conceived and designed the study. K.P. performed the experiments. All authors contributed to the analysis and interpretation of the results and writing of the manuscript. K.P., B.B. and V.B. performed the ChIP-exo experiments, B.Z. analyzed the data. A.V. Modelled the MALR complexes with DNA. K.P. and F.A.K. conceived the evolutionary model and carried out corresponding bioinformatic analyses.

## Additional information

**Accession Codes.** The sequences generated in the ChIP-exo experiment have been deposited in Gene Expression Omnibus database under the accession code GSE57902.

**How to cite this article:** Pougach, K. *et al.* Duplication of a promiscuous transcription factor drives the emergence of a new regulatory network. *Nat. Commun.* 5:4868 doi: 10.1038/ncomms5868 (2014).

## Supplementary Material

Supplementary Figures and Supplementary Reference Supplementary Figures 1-5 and Supplementary Reference

Supplementary Data 1 Transcription factor binding sites found in the promoters of maltose- or palatinose-specific genes in *S. cerevisiae, S. kudryavzevii, S. bayanus, S. mikatae, S. paradoxus.* Gene and species names are listed in the first column; transcription factor binding sites (TFBS) found in the promoter regions of these genes are listed in the columns 2, 3 and 4. The last 4 or 3 digits of the corresponding contig number are used as gene identifiers in species other then *S. cerevisiae.* Maltose-specific genes and CGC-containing (Malx3 like) binding sites are highlighted in red, palatinose-specific genes and CGG-containing (Yfl052w like) binding sites are highlighted in blue.

Supplementary Data 2 Strain and primer list. Sheet 1 contains a full strain list with complete details about strain construction. Sheet 2 lists all primers used for strain construction.
