# Supplementary material for: Duplication of a promiscuous transcription factor drives the emergence of a new regulatory network
Source: Nat Commun. 2014 Sep 10;5:4868. doi: 10.1038/ncomms5868 (PMC4172970; doi:10.1038/ncomms5868)
Supplement: Supplementary Figures and Supplementary Reference — Supplementary Figures 1-5 and Supplementary Reference [file ncomms5868-s1.pdf]

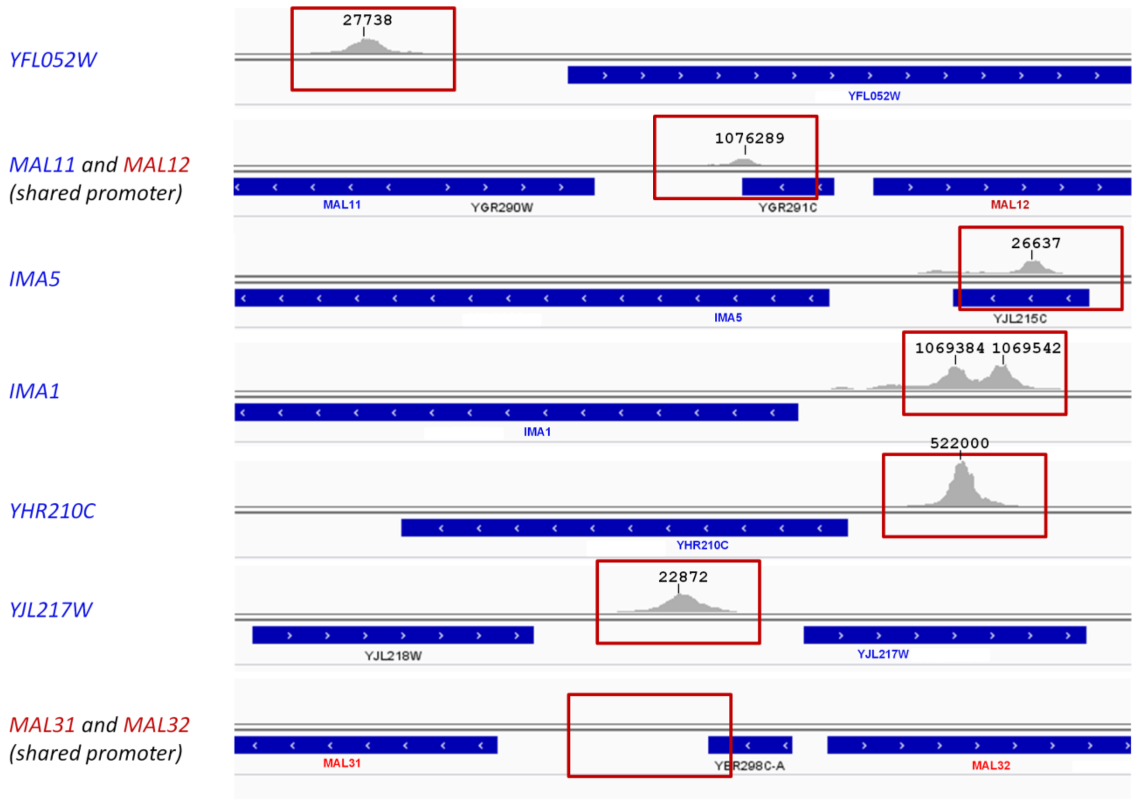

Supplementary figure 1. ChIP-exo analysis reveals Yfl052w binding sites. Raw ChIP-exo reads from one of the two duplicated experiments are shown in IGV viewer for each gene of interest, scaled [min=0, max=25000 reads]. Chromosomal coordinate of each Yfl052w binding site coming from the comprehensive bioinformatic analysis of peak pairs is indicated on top of each peak. The promoter region of each gene of interest is encircled in red. Note that this figure shows the mapped raw reads. Further data processing enables precise identification of the binding motifs[1].

SUPPLEMENTARY FIGURE 2 VERSTREPEN

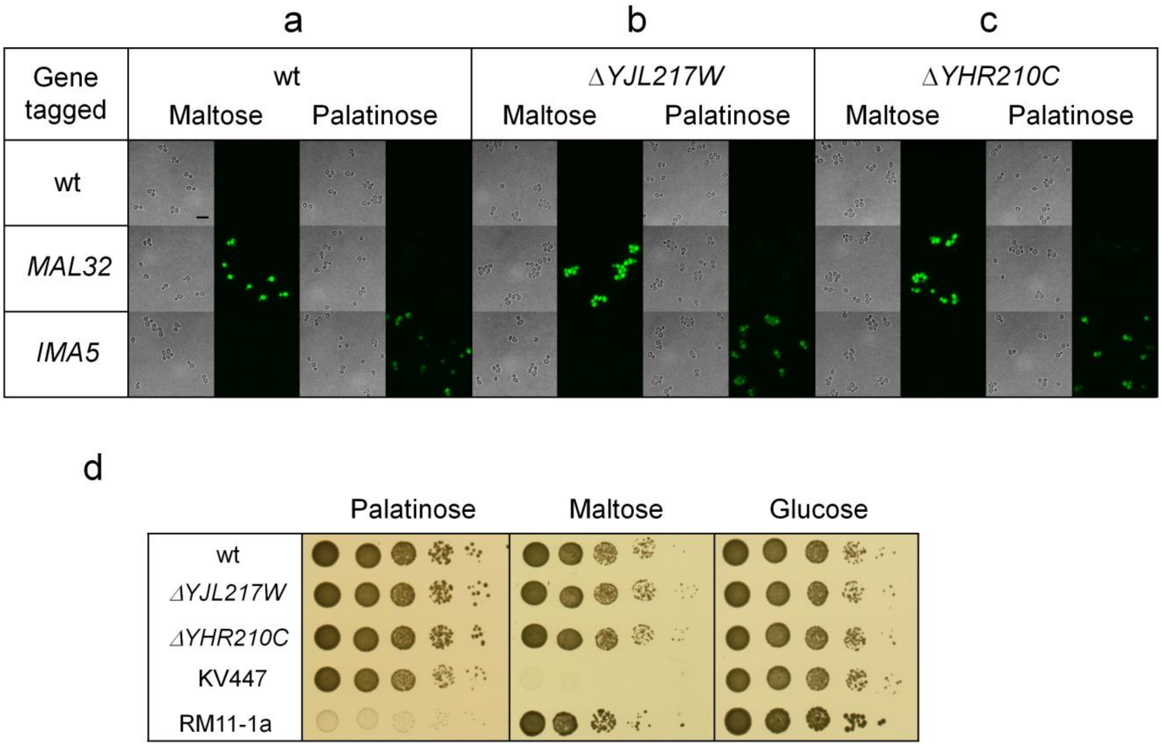

Supplementary figure 2. The deletion of noncanonical targets of Yfl052w does not affect cell growth or gene expression in maltose or palatinose. Representative brightfield and fluorescence microscopy images of yeast cells with *MAL32* or *IMA5* genes fluorescently tagged are shown for wt cells (**a**), and strains carrying deletions of *YJL217W* or *YHR210C* (panel **b** and panel **c**, respectively). Cells were grown in presence of either palatinose (α 1-6 disaccharide) or maltose (α 1-4 disaccharide) as indicated above the pictures. Scale bar is included in the upper left image and equals 10 μm. (**d**) Growth profiles of the wild type cells and strains carrying deletions of *YJL217W* or *YHR210C* determined by spot assays on YP plates containing the indicated sugar and the inhibitor of respiration (antimycin A). Cells incapable of fermenting respective sugar show no growth on such medium. Laboratory strains KV447 and RM11-1a are used as a control (KV447 is incapable of fermenting maltose, RM11-1a is incapable of fermenting palatinose).

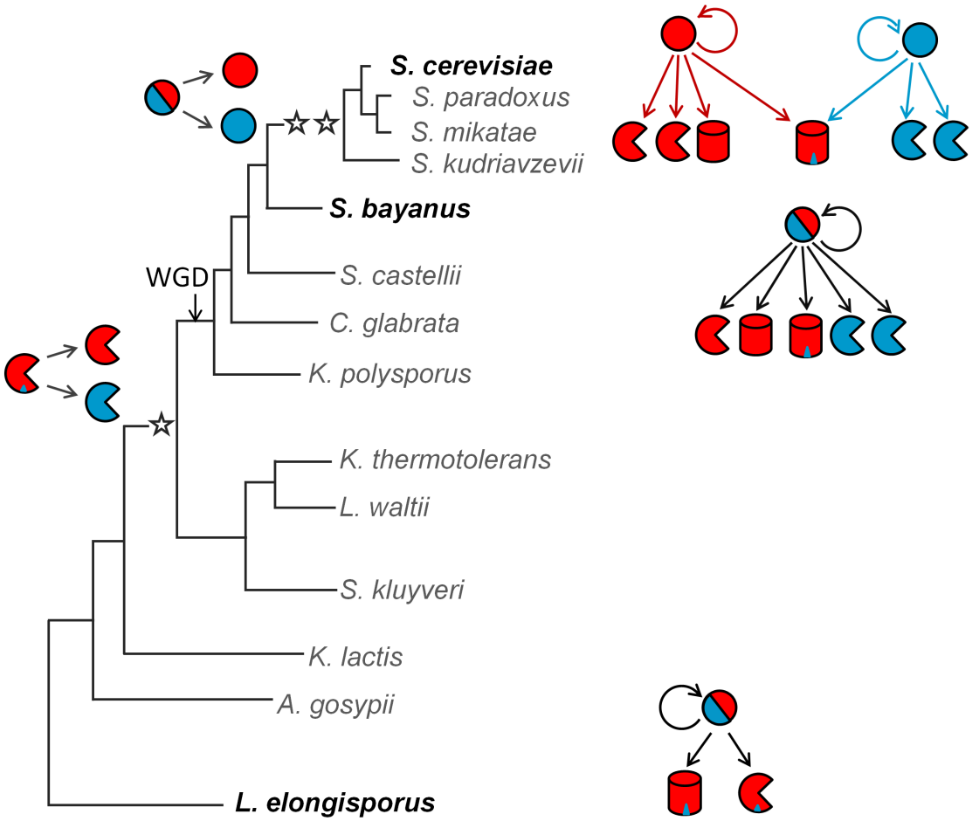

Supplementary figure 3. Duplication and specialization of MalS enzymes preceded that of MalR regulators. Yeast phylogenetic tree showing the key evolutionary events of the *MALS* and *MALT* genes (*duplication and neofunctionalization of *MALS* genes and **duplication and diversification of *MALR* genes). *MALR* genes are depicted in a shape of a circle; *MALT* genes are represented as barrels, *MALS* genes – three-quarter pies. Blue color indicates specificity of the corresponding protein to the palatinose, red – to maltose. *MAL* regulatory networks present in the highlighted species are shown on the right. WGD – whole-genome duplication.

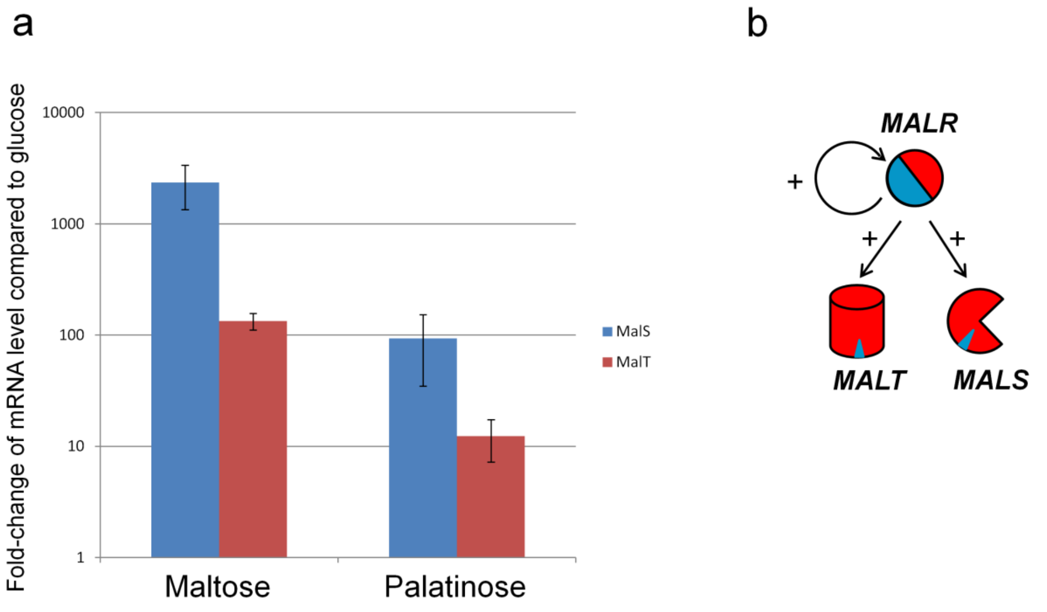

Supplementary figure 4. MAL regulatory network in *L. elongisporus* possibly resembles that of pre-duplication ancestror. (**a**) Relative expression levels of *MALS* and *MALT* from *L. elongisporus* in maltose and palatinose compared to glucose. mRNA levels were normalized to actin mRNA, which shows no changes in expression under the conditions used. Error bars indicate SD derived from two independent biological replicates. (**b**) The most likely *MAL* gene regulatory network of *L. elongisporus,* with the sole MalR regulator inducing the transcription of the promiscuous *MALS* and *MALT* genes in response to both α 1-4 and α 1-6 disaccharides.

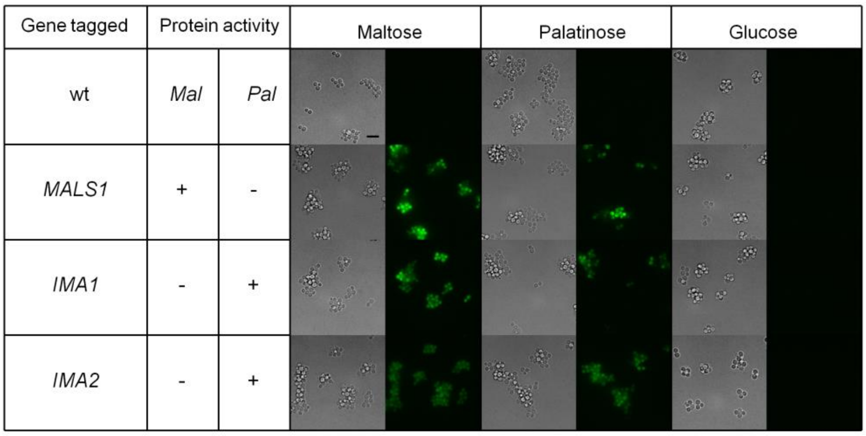

Supplementary figure 5. Maltose and palatinose-specific *MALS* genes in *S. bayanus* are activated in presence of both maltose and palatinose. Representative brightfield and fluorescence microscopy images of *S. bayanus* cells with various *MALS* or *IMA* genes tagged with a fluorescent reporter gene yECitrine. Cells were grown in either maltose, palatinose or glucose. Scale bar is included in the upper left image and equals 10 μm.

Supplementary references

1. Rhee, H. S. & Pugh, B. F. Comprehensive genome-wide protein-DNA interactions detected at single-nucleotide resolution. *Cell* **147,** 1408–19 (2011).
